# HaNDL syndrome: a reversible cerebral vasoconstriction triggered by an infection? A case report and a case-based review

**DOI:** 10.1186/s40001-022-00815-8

**Published:** 2022-10-08

**Authors:** Giuseppe Fiamingo, Isabella Canavero, Matteo Gastaldi, Elisa Coloberti, Gabriele Buongarzone, Natascia Ghiotto, Ana Bacila, Alfredo Costa, Sabrina Ravaglia

**Affiliations:** 1 Department of Brain and Behavioral Sciences, University of Pavia, Pavia, Italy; 2 IRCCS Mondino Foundation, Via Mondino 2, 27100 Pavia, Italy; 3 Cerebrovascular Unit, Fondazione IRCCS Istituto Neurologico Carlo Besta, Milan, Italy

**Keywords:** RCVS reversible cerebral vasoconstriction, Nimodipine, Headache, Corpus callosum, EBV, HaNDL

## Abstract

**Background:**

The syndrome of transient Headache and Neurological Deficits with cerebrospinal fluid (CSF) Lymphocytosis (HaNDL) is classified among secondary headaches attributed to “non-infectious, inflammatory intracranial disease”. Despite its classification among secondary headaches, the current definition of HaNDL does not contemplate a causal agent. Thus, the aetiology, as well as the pathogenesis of both the headache and the transient focal deficits, remains unknown.

**Case presentation:**

We describe a 29-year-old healthy male developing episodes of thunderclap headaches associated with recurrence of hemiparesis/hemi-paraesthesia; CSF showed lymphocytosis 200/mm^3^ and increased albumin; brain MRI revealed widespread leptomeningeal enhancement and a non-enhancing, circular diffusion restriction in the splenium of corpus callosum. Screening for neurotropic pathogens detected Epstein-Barr (EBV) DNA in serum and CSF, interpreted as a primary EBV infection once the seroconversion of EBV nuclear antigen (EBNA) IgM to IgG was proven on follow-up. Transcranial Doppler detected, during headache, increased flow velocity in middle cerebral arteries, possibly indicating vasospasm. Oral nimodipine was administered, with prompt clinical recovery, resolution of CSF/MRI abnormalities, and normalization of flow velocities in middle cerebral arteries.

**Case-based review:**

Although the definition of HaNDL does not contemplate a viral trigger or abnormal brain imaging, we found other literature cases of HaNDL associated with direct or indirect signs of CNS infection.

**Conclusions:**

At least in a proportion of patients, a viral aetiology may have a role in HaNDL. Whatever the aetiology, we suggest that the pathogenic mechanism may rely on the (viral or other) agent ultimately triggering cerebral vasoconstriction, which would explain both focal symptoms and headache. Calcium channel blockers might be a therapeutic option.

## Background

The syndrome of transient Headache and Neurological Deficits with cerebrospinal fluid Lymphocytosis (HaNDL) is characterized by migraine-like headaches accompanied by transient neurological deficits, most frequently hemi-paraesthesia, hemiparesis, and dysphasia; unlike classical migraine with aura, visual symptoms are uncommon [1]. The clinical course is benign and self-limiting, with 1–12 attacks of several hours’ duration (usually > 4 h), [2, 3] recurring over a one-to-three-month period. Diagnosis relies on the exclusion of alternative causes; thus, MRI is claimed as normal.

To date, the aetiology of HaNDL remains undetermined: this is somewhat contradictory, considering its classification among secondary headaches [1]. Since the hallmark of the syndrome is cerebrospinal fluid (CSF) lymphocytic pleocytosis (> 15/mL) [1], extensive screening for infections is generally performed, usually with negative findings. The pathophysiology of the transient neurological deficits accompanying headache is also undetermined [1, 2]: cortical spreading depression (CSD) has been hypothesized, but never demonstrated; actually, the features of the neurological deficits associated with HaNDL appear distinct from migraine auras both in pattern and duration.

Literature reports on HaNDL are limited to few isolated cases or very small series, so that a systematic assessment of clinical, laboratory and imaging features is lacking. It is also likely that HaNDL is an under-recognized condition, since patients with concomitant vascular risk factors may be misdiagnosed as strokes: indeed, patients receiving potentially harmful treatments, even thrombolysis, have been reported [4, 5].

We hereby report a patient fulfilling the clinical inclusion criteria for HaNDL [1], but with abnormal brain MRI (widespread leptomeningeal enhancement, oedema-like corpus callosum lesion). Extensive screening for infections revealed a self-limiting primary EBV infection, without clinically relevant signs or symptoms of meningoencephalitis. Based on neurosonology suggesting cerebral vasoconstriction, we decided to treat the patient with nimodipine, as borrowed from reversible cerebral vasoconstriction syndromes (RCVS). Since both MRI abnormalities and infections are considered as exclusion criteria for the diagnosis of HaNDL, we revised all literature cases of HaNDL, collecting those with positive brain MRI, positive infective screening, or abnormal findings in intracranial vessels; we then discuss potential controversies in diagnosis, misdiagnosis, and potential etiopathogenetic hypotheses.

## Case presentation

A 29-year-old male with no history of headache referred to the Emergency Department for two episodes of sudden-onset, maximal-intensity posterior bilateral headache associated with nausea and hemi-paraesthesia, ascending from the left lower limb up to the face, followed by ipsilateral hemiparesis. Both episodes completely recovered within 2 h. In the previous 2 weeks, he had complained of fatigue, insomnia, and migraine-like headaches progressively worsening in intensity and frequency. Neurological evaluation and non-contrast brain CT were unremarkable. He was discharged with a diagnosis of migraine with aura.

Three days later, he was admitted to our Emergency Neurology Unit for a third episode.

**Fig. 1:**
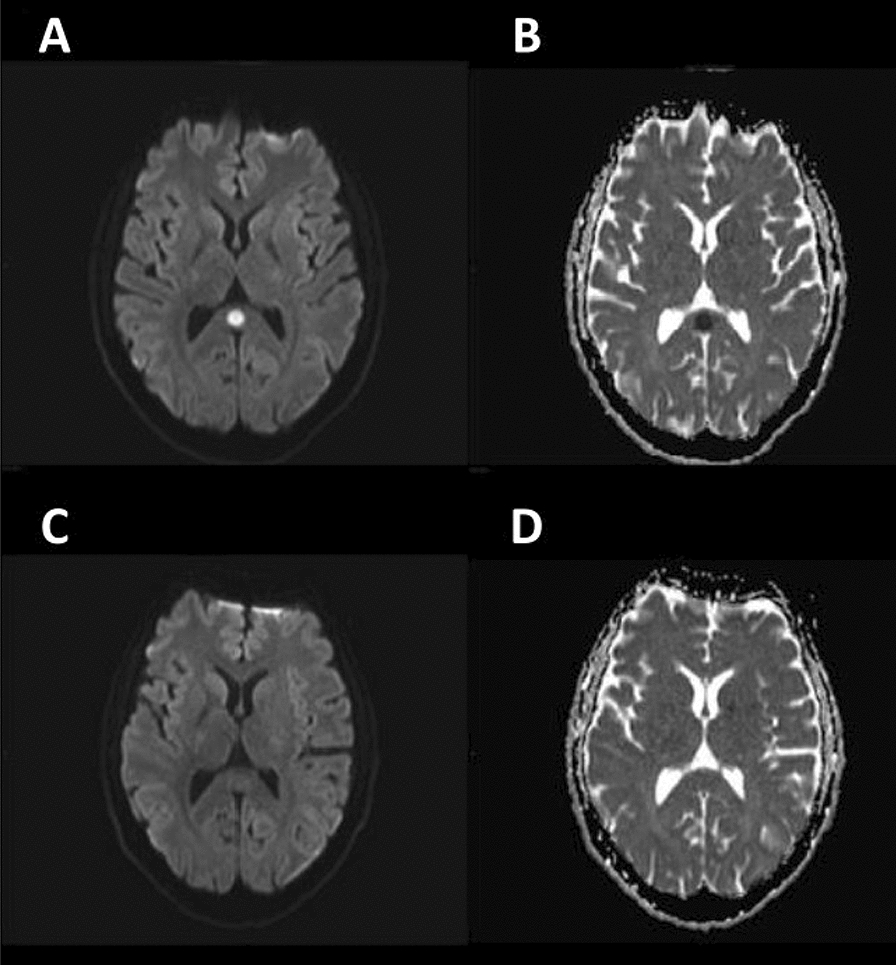
CLOCC. Axial Brain MRI with non-enhancing, midline circular hyperintensity in DWI **A** and hypointensity in ADC map **B** in the corpus callosum, suggesting oedema, compatible with CLOCC (“Cytotoxic Lesion Of the Corpus Callosum”). 10 days after, MRI shows complete resolution of the lesion **C** and **D**

**Fig. 2:**
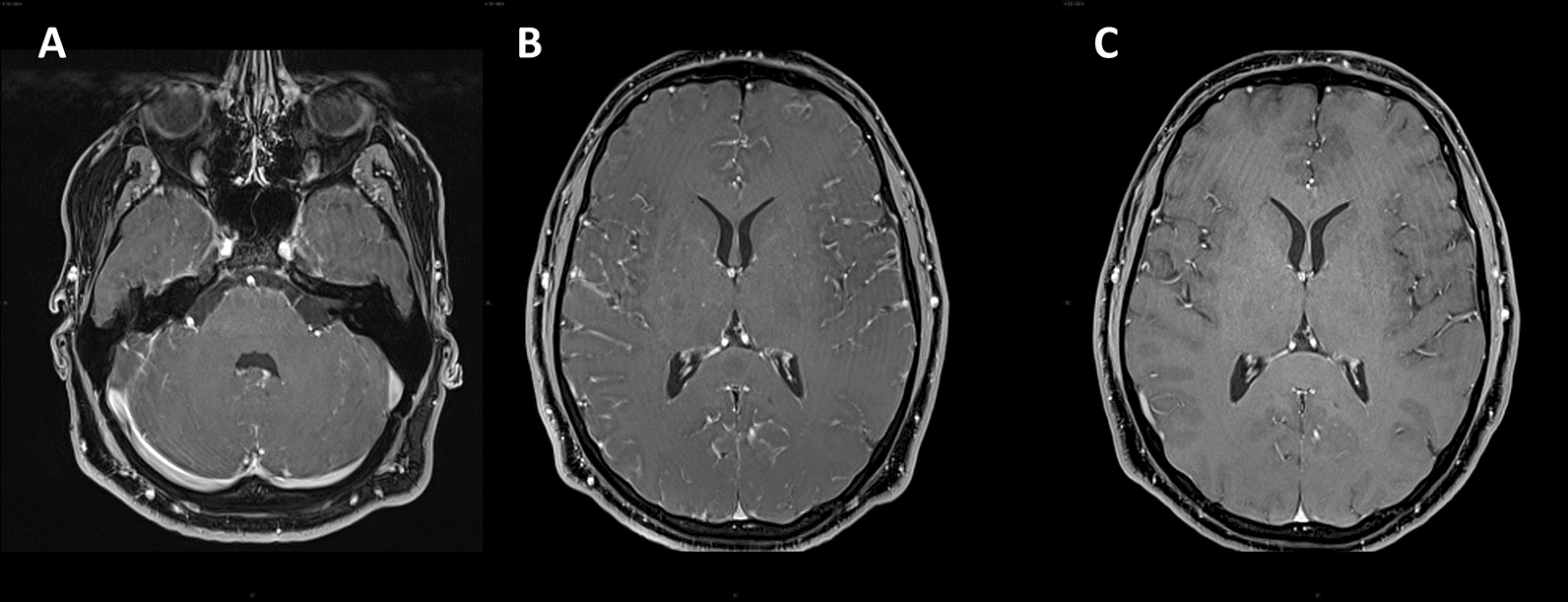
MRI shows diffuse leptomeningeal enhancement (subtentorial, **A**; supratentorial, **B**); Figure **C** shows resolution of leptomeningeal enhancement on follow-up MRI

Within the first hours from admission, he experienced five thunderclap headache attacks, two of which were associated with a 4-h-duration left hemiparesis. Blood pressure was normal. A RCVS was hypothesized: the patient was administered oral nimodipine 60 mg, followed by 30 mg every 4 h, with prompt symptoms resolution. Brain MRI revealed a circular diffusion restriction in the splenium of corpus callosum and diffuse leptomeningeal (but not callosal) enhancement (Figs 1, 2). MR angiography, MRI spectroscopy, and perfusion-weighted imaging were normal. CSF showed lymphocytic pleocytosis (200/mm^3^), 6.5% CSF/serum albumin ratio (normal values: < 0.7%), and a mirror serum/CSF band pattern. Blood and CSF angiotensin-converting enzyme was negative. Search for neurotropic pathogens was negative, except for positive EBV-DNA PCR in both serum (5310 copies/mL) and CSF (2620 copies/mL), with positive EBV VCA IgG, and negative VCA-IgM, EBNA-IgM, and EBNA-IgG**.** CSF/blood immunophenotyping did not reveal blasts/cancer cells. Contrast-enhanced body CT revealed mild spleen enlargement. EEG showed bilateral diffuse anterior theta-delta slowing, without epileptiform abnormalities. Transcranial Doppler (TCD), performed during the third day of nimodipine treatment, revealed increased mean flow velocity in Middle Cerebral Arteries (MCA-MFV), not reaching the vasospasm threshold (right MCA: MFV 91 cm/s, peak 137 cm/s; left MCA: MFV 86 cm/s, peak 127 cm/s normal values: MCA-MFV < 80 cm/s, MCA peak < 120 cm/s) (Table 1). During hospitalization, no relapses occurred for both headache and focal symptoms. Nimodipine was tapered to a maintenance dose of 30 mg every 8 h for 6 weeks. Follow-up CSF analysis on the 7th day revealed a marked reduction in cells (60/mm^3^) and CSF/blood albumin ratio (2.9%). 10 days after admission, follow-up brain and spine MRI was normal (Figs. 1, 2). Follow-up TCD showed normal MCA-MFV. The patient was discharged with complete resolution. After 1 month, EBV serology revealed EBNA seroconversion with positive EBNA-IgG index (8.0, range 0.2–0.8, Multiplex Luminex Method), supporting that a primary EBV infection had occurred.

**Table 1:** HaNDL syndrome cases with either positive infectivologic screening or MRI abnormalities

	Patient (age, sex)	CSF (WBC–protein)	Blood serology	CSF serology/PCR	MRI	TCD–MCA-MFV^a^	Therapy
Emond et al. 2009 [6]	18 y F	350/μl–200 mg/dl	**IgM HHV-6, slight increase CMV IgM**	Negative	**LE**	–	Acyclovir 10 mg/kg/bid/14 days + ceftriaxone 2 g/bid/3 days
Yilmaz et al. 2010 [28]	27 y F	60/μl–150 mg/dl	Negative	Negative	**Focal LE**	–	Verapamil 240 mg/day, prednisolone 50 mg/day, valproic acid 1000 mg/day
Raets 2012 [15]	32 y M	123/μl–80 mg/dl	–	Negative	**CLOCC**	–	Paracetamol
Goncalves et al. 2013 [29]	14 y F	250/μl–normal	Negative	Negative	**Focal LE**	–	Acyclovir
Apetse et al. 2013 [9]	19 y M	250/μl–54 mg/dl	**IgM VCA EBV, no 1-month seroconversion**	Negative	Normal	–	Acyclovir 10 mg/kg/8 h
Filina et al. 2013 [30]	16 y M	110/μl–83 mg/dl	Negative	Negative	**LE**	–	Acyclovir, ceftriaxone, vancomycin
Stelten et al. 2016 [7]	23 y F	220/μl–143 mg/dl	**IgG HHV-6–7**	**HHV-7**	Normal	–	Acyclovir 10 mg/kg/8 h, methylprednisolone 1 g/5 days
Babi et al. 2017 [31]	31 y M	625/μl–325 mg/dl	Negative	Negative	**LE**	–	Acyclovir, vancomycin, ceftriaxone, and ampicillin, acetazolamide 1000 mg/day
Verentiotzi et al. 2017 [10]	28 y M	316 μl–174 mg/dl	**IgM IgG CMV**	Negative	Normal	–	–
Patel et al. 2017 [20]	25 y M	162/μl–245 mg/dl	–	Negative	**LE**	–	–
García-Esperón et al. 2017 [32]	30 y M	92/μl–145 mg/dl	Negative	Negative	**Focal LE**	Normal	–
Armstrong-Javors et al. 2019 [3]	16 y M	303/μl–131 mg/dl	negative	Negative	**LE**	–	Sumatriptan, metoclopramide, ketorolac, acetazolamide
Vieira et al. 2019 [11]	15 y M	194/μl–53 mg/dl	Negative	**Borrelia lusitaniae**	normal	–	(Acyclovir + ceftriaxone)/21 days
Rodríguez-López et al. 2019 [33]	41 y M	40/μl–186 mg/dl	Negative	Negative	**LE**	–	NSAID, Corticosteroids
Smail et al. 2020 [14]	47 y M	89/μl–87 mg/dl	Negative	Negative	**CLOCC**	–	–
Sisman et al. 2020 [16]	33 y M	13/μl–146 mg/dl	Negative	Negative	**CLOCC**	–	–
Sànchez-Miranda Romàn et al. 2021 [8]	31 y M	150/μl–131 mg/dl	Negative	**HHV-7**	**LE**	–	Acyclovir, corticosteroids
Our patient	29 y M	200/μl–281 mg/dl	**EBV-DNA, EBV VCA IgG, 1-month EBNA-IgG seroconversion**	**EBV-DNA**	**CLOCC and LE**	L 86 cm/s R 91 cm/s	Oral nimodipine 60 statim, 30 mg/6 h/3 months
Kappler et al. 1997 [23]	32 y M	109/μl–130 mg/dl	Negative	Negative	Normal	L 114 cm/s R 56 cm/s	Dexamethasone 48 mg/bid/5 days
Kappler et al. 1997 [23]	29 y M	107/μl–71 mg/dl	Negative	Negative	Normal	L 38 cm/s R 80 cm/s	Dexamethasone 48 mg/bid/5 days
Serrano-Castro et al. 2000 [24]	27 y M	210/ul–74 mg/dl	Negative	Negative	Normal	L 46 cm/s R 59 cm/s (+ instability?)	Ceftriaxone iv/10 days
de la Cruz et al. 2019 [25]	42 y M	40/μl–100 mg/dl	Negative	Negative	Normal	**142 cm/s bilaterally**	Aciclovir 10 mg/kg/8 h/5 days, iv nimodipine

The last four rows refer to literature reports mentioning TCD findings

^a^MCA-MFV: normal values: 44–76; upper limits of normal: 77–85; high: > 85; vasospasm: > 120 cm/s

*M* male, *F* female, *CSF* cerebrospinal fluid, *WBC* white blood cells, *MRI* magnetic resonance imaging, *TCD *trans cranial Doppler, *CMV* cytomegalovirus, *EBV* Epstein–Barr virus, *HHV *human herpesvirus, *CLOCC* cytotoxic lesion of corpus callosum, *LE* leptomeningeal enhancement, *MCA*-*MFV* middle cerebral artery mean flow velocity, *L* left, *R* right

### Literature review: search strategy

Studies were identified by Pubmed, by entering the keywords: pseudo-migraine, headache and neurological deficits with cerebrospinal fluid lymphocytosis, headache and pleocytosis, and HaNDL. The search yielded 134 case reports and two small series, including a literature review on the paediatric population. Studies in English and Spanish language were included. The electronic search was supplemented by a manual search of references and reviews. Two Authors (GF and GB) independently screened full texts, to verify whether the patients fulfilled clinical inclusion criteria for HaNDL. Among patients with HaNDL, we selected those with (a) positive screening for infections OR (b) positive brain imaging. Unfortunately, many reports do not specify which infections were screened for, or whether MRI was contrast enhanced or not; in a few reports MRI was not performed and exclusion of alternative aetiologies relied on CT angiography.

We defined as “positive brain imaging” those abnormalities detected by conventional MRI. We excluded abnormalities detected by advanced imaging techniques (perfusion SPECT/CT, perfusion MRI), due to uncertain interpretation of findings: for instance, documentation of altered perfusion may be cause or consequence, and usually does not explain the primary mechanism. The extracted data were as follows: first Author, year of publication, patient’s age, sex, CSF features, results of blood serology or CSF serology/genome search, and results of brain MRI. We recorded the chosen treatment, but did not extrapolate any judgement about treatment effectiveness.

We thus could retry a total amount of 18 reports, 10 including MRI abnormalities only (focal or widespread leptomeningeal enhancement: *n* = 8; callosal oedema: *n* = 2), 4 with positive screening for infections, and 2 with both infection and MRI abnormalities. We also annotated the reports in which the Authors, in view of the transient focal deficits, decided to perform transcranial Doppler (*n* = 3).

The results are in Table 1, where also the current case is reported for comparison.

## Discussion

The differential diagnosis of HaNDL is challenging, due to its mimic of cerebrovascular disorders. Moreover, the condition is not widely known, even among neurologists (especially those not exclusively dedicated to headache), and even less understood. The unclear etiopathogenesis does not help strengthen our interest in this condition: its very classification among secondary headaches, in spite of the lack of a known causal agent, is somewhat ambiguous. Criteria for HaNDL include clinical (headache and transient neurological deficits) as well as laboratory features (CSF pleocytosis), and both these features do not have an explanation. Brain MRI and screening for infections are claimed as negative, so that HaNDL is classified among “non-infectious” diseases, “with undetermined aetiology and pathogenesis” [1]. Thus, more than defining what it is, the International Classification of headache Disorders (ICHD) defines what it is not. In our patient, a clinical/CSF picture consistent with HaNDL co-occurred with a primary EBV infection, with imaging findings also supporting an infectious trigger.

An epileptic aetiology was considered in the differential diagnosis of the transient, recurrent, and somewhat stereotyped focal neurological deficits observed in our patient. However, he underwent two routine EEGs in close temporal relationship with the focal symptoms, without evidence of interictal epileptic discharges or residual focal slowing; the clinical picture was quite abrupt in onset, and characterized by “negative” symptoms; during the episodes, the patient was always alert and oriented; the 2–4 h’ duration without changes in the clinical picture seems quite too long for a focal epilepsy.

### Aetiologic clues

#### 1. Primary EBV infection

In our patient, we detected EBV-DNA genome in serum > CSF: although this is often regarded as a non-specific reactivation, follow-up evidence of seroconversion confirmed that a primary EBV infection had occurred; indeed, the patient had mild signs/symptoms reminiscent of mononucleosis (malaise, fatigue, mild spleen enlargement).

Although the screening for infections is usually claimed as negative in HaNDL [1, 2], most reports do not specify which agents were searched for; of note, a viral prodrome is reported in 25–50% of patients [3], possibly suggesting, if not a concomitant infection, at least an infectious trigger; the self-limiting disease course, with spontaneous resolution over one-to–three months, is also in line with this hypothesis. Few cases of infection during HaNDL are reported (Table 1), including HHV-6/-7 [6–8], EBV [9], CMV [6, 10], and Borrelia [11]. As regards EBV [9], increased IgM-EBV VCA is mentioned, without seroconversion on follow-up serology, thus suggesting, unlike our case, a non-specific reactivation, rather than a primary EBV infection.

Actually, our patient did not show a clinical picture fully convincing for a CNS involvement by EBV, since (a) he did not show the classical features of meningitis (headache was episodic, not persistent, and associated with focal neurological dysfunction; he lacked photophobia, neck stiffness, and fever > 37 °C) or encephalitis (mental state was normal, there was no somnolence or confusion, neurological signs were focal, not diffuse, and recurrent, but not persistent); (b) he recovered completely and rapidly without antiviral therapy. Moreover, EBV is rarely associated with meningoencephalitis in immunocompetent patients: the patient did not show current or anamnestic clues to immunodeficiency, HIV infection was excluded, and cytofluorometry did not show abnormalities in white blood cells.

Our case-based literature review could detect at least six other cases associated with an infectious trigger [6–11]. Unfortunately, we cannot say which proportion of the total cases they represent, since details on infectious screening are particularly scarce, most reports lacking information about which infections were searched for, the timing of this search, or whether the screening was performed at all.

However, some indirect clues to a concomitant infection may also come from the evaluation of MRI abnormalities.

#### 2. MRI abnormalities

Our patient showed leptomeningeal enhancement and a transient oedema-like lesion of the corpus callosum, consistent with a “cytotoxic lesion of the corpus callosum” (CLOCCs) [12]. We believe that both findings indirectly support a CNS inflammation

- CLOCC: First, CLOCCs are considered as purely MRI markers without a clear clinical correlate, accompanying several conditions that ultimately lead to cytokines release, increased blood–brain barrier (BBB) permeability, perivascular/intramyelinic oedema, inflammatory infiltrates, and cytotoxic oedema [12]. As many as one-third of patients with CLOCC recognize, among triggers, viral infections (either of the CSF or systemic), including EBV [13].

In our patient, the association of HaNDL with CLOCC suggests a shared aetiology between the two conditions, and the pathogenic link could be an infection. There are three other reports of an association between HaNDL and CLOCC [14–16] (Table 1), all failing to detect infections. Again, the Authors did not specify the timing of CSF analysis and which agents were searched for. Indeed, in one patient, CSF showed a mirror CSF/serum band pattern [14], that we also observed in our patient, and that may suggest an infectious aetiology (Table 1).

The finding of CLOCC in patients with migraine with aura, with aura manifesting as isolated paresthesias without the typical visual disturbances [17–19], may also have masked a HaNDL syndrome. However, CSF analysis was performed only in one case [17], likely because the diagnosis of HaNDL was not taken into account.

b) Leptomeningeal enhancement: Unlike previous cases of HaNDL with CLOCC, our patient also showed diffuse and symmetric leptomeningeal enhancement, a finding typically seen in viral meningitis. Leptomeningeal enhancement has also been reported in HaNDL [3, 6, 20] supporting an infection-triggered inflammatory mechanism, at least when a concurrent infection cannot be ascertained: indeed, in one-third of presumed viral meningitis the agent remains unknown [21].

### Pathogenic clues

#### 3. Vasospasm

The mechanism of transient neurological dysfunction in HaNDL is also unknown, with vasomotor changes versus Cortical Spreading Depression (CSD) as the most plausible hypotheses. Case reports on perfusion SPECT/MRI, trying to clarify this point, seem to support the CSD mechanism, but with inconclusive or controversial results. The techniques were, however, unable to establish whether hypoperfusion is cause or consequence [5, 22].

In our patient, we support the occurrence of a cerebral vascular damage either caused directly by EBV infection or by its triggering an immune-mediated, post-, or para-infectious mechanism. Headache features, ultrasound findings, and an apparent response to nimodipine all seem to suggest a role for vasoconstriction in the pathogenesis of his transient neurological symptoms and headache. We did not perform catheter angiography, which is the gold standard assessment for focal cerebral vasoconstriction related to RCVS, as the patient had a spontaneously favourable clinical outcome. However, the TCD, although performed three days after starting nimodipine and during symptoms remission, still showed bilateral MCA-MFV acceleration. Despite the occurrence of transient focal deficits, surprisingly few studies have described TCD use to explore possible underlying vasomotor changes in HaNDL during or after the attacks. The only three available reports describe: asymmetrical elevation or reduction of MCA-MFV on the symptomatic side [23]; high MCA-VMF instability in different settings [24]; and true vasospasm [25] (Table 1). The heterogeneous TCD patterns may be related to the timing of examination. The hypothesis of multifocal but isolated episodes of intracranial vasomotor changes, rather than a CSD mechanism, is also suggested by the pattern of focal symptoms in HaNDL, characterized by the unexpected paucity of visual “aura”, that is also in line with the other cases reported in literature. The duration of transient focal symptoms (2–4 h in our patient) further supports a phenomenon distinct from migraine aura. Moreover, the apparent clinical response to nimodipine suggest a role for cerebral vasoconstriction. HaNDL is a self-limiting disease and thus we cannot exclude spontaneous recovery, but the prompt response, as well as the shorter time course of the whole syndrome (< 1 month in our patient vs 1–3 months in literature cases), both support an effect of the treatment.

Nimodipine was also used in the sole TCD study showing bilateral cerebral vasospasm in HaNDL [25]: unfortunately, its effectiveness could not be ascertained, since the patient described in this report had required deep sedation during hospitalization.

Two other reports support this vasomotor hypothesis, by linking HaNDL to autoimmunity against voltage-gated calcium channels (VGCC) [26, 27]. Accordingly, nimodipine administration appeared to be reasonably effective, as the drug antagonized the transiently increased VGCC function.

## Conclusions

HaNDL is classified among secondary headaches attributed to “non-infectious inflammatory intracranial disease” [1]. Despite evidence of EBV infection and MRI abnormalities, we labelled our patient’s syndrome as HaNDL, which is usually a diagnosis of exclusion. Indeed, sometimes exceptions and rare manifestations allow speculating correctly on the etiopathogenesis of “idiopathic” diseases. By reviewing literature reports, we found similar cases with MRI indirectly supporting an infection or with direct evidence of positive infectious screening. We thus believe that, at least in some cases, HaNDL may have a post- or para-infectious immune-mediated aetiology, and that detection of an associated infection should not categorically represent an exclusion criterion. Whether EBV or other agents are directly pathogenic on small vessels or cause a transient autoimmune vasculitis remains speculative. In our patient, CNS signs of EBV infection were not fully convincing or at least not clinically relevant, but the infection could have triggered a direct or immune-mediated, self-limiting, vascular damage leading to cerebral vasoconstriction, and explaining both focal deficits and headache. Further evidence is warranted to support the role of cerebral vasoconstriction and to prove the potential therapeutic role of calcium channel inhibitors.

We also believe that HaNDL could be underestimated, and better knowledge of this condition among general neurologists, especially those working in emergency settings and who are daily facing its mimics (strokes and newly onset headaches), would help in recognition of further cases. Improved awareness would also help collect more cases during the acute phase of disease, implement a screening for infections, and use transcranial neurosonology to help clarify the source of headache and transient deficits.

## Data Availability

Data sharing is not applicable to this article as no datasets were generated or analysed during the current study.

## Abbreviations

BBB: Blood–brain barrier
CLOCC: Cytotoxic lesion of the corpus callosum
CMV: Cytomegalovirus
CNS: Central nervous system
CSD: Cortical spreading depression
CSF: Cerebrospinal fluid
CT: Computed tomography
EBNA: Epstein–Barr nuclear antigen
EBV: Epstein–Barr Virus
EEG: Electroencephalography
HaNDL: Headache and Neurological Deficits with cerebrospinal fluid Lymphocytosis
HHV: Human Herpes Virus
ICHD: International Classification of Headache Disorders
MCA-MFV: Middle cerebral arteries mean flow velocity
MRI: Magnetic resonance imaging
PCR: Polymerase chain reaction
RCVS: Reversible cerebral vasoconstriction syndrome
SPECT: Single-photon emission computed tomography
TCD: Transcranial Doppler
TH: Thunderclap headache
VCA: Viral capsid antigen
VGCC: Voltage-gated calcium channel
